# Focus on Extracellular Vesicles: Therapeutic Potential of Stem Cell-Derived Extracellular Vesicles

**DOI:** 10.3390/ijms17020174

**Published:** 2016-02-06

**Authors:** Bin Zhang, Ronne Wee Yeh Yeo, Kok Hian Tan, Sai Kiang Lim

**Affiliations:** 1 Exosome and Secreted Nano-vesicle Group, A*STAR Institute of Medical Biology, 8A Biomedical Grove, #05-05 Immunos, Singapore 138648, Singapore; bin.zhang@imb.a-star.edu.sg (B.Z.); ronne.yeo@imb.a-star.edu.sg (R.W.Y.Y.); 2 Department of Maternal Fetal Medicine, KK Women’s and Children’s Hospital, 100 Bukit Timah Road Women’s Tower, Level 3, Singapore 229899, Singapore; tan.kok.hian@kkh.com.sg; 3 Department of Surgery, YLL School of Medicine, National University of Singapore C/O NUHS Tower Block, Level 8, IE Kent Ridge Road, Singapore 119228, Singapore

**Keywords:** stem cells, extracellular vesicles, exosomes, cellular regenerative therapeutics

## Abstract

The intense research focus on stem and progenitor cells could be attributed to their differentiation potential to generate new cells to replace diseased or lost cells in many highly intractable degenerative diseases, such as Alzheimer disease, multiple sclerosis, and heart diseases. However, experimental and clinical studies have increasingly attributed the therapeutic efficacy of these cells to their secretion. While stem and progenitor cells secreted many therapeutic molecules, none of these molecules singly or in combination could recapitulate the functional effects of stem cell transplantations. Recently, it was reported that extracellular vesicles (EVs) could recapitulate the therapeutic effects of stem cell transplantation. Based on the observations reported thus far, the prevailing hypothesis is that stem cell EVs exert their therapeutic effects by transferring biologically active molecules such as proteins, lipids, mRNA, and microRNA from the stem cells to injured or diseased cells. In this respect, stem cell EVs are similar to EVs from other cell types. They are both primarily vehicles for intercellular communication. Therefore, the differentiating factor is likely due to the composition of their cargo. The cargo of EVs from different cell types are known to include a common set of proteins and also proteins that reflect the cell source of the EVs and the physiological or pathological state of the cell source. Hence, elucidation of the stem cell EV cargo would provide an insight into the multiple physiological or biochemical changes necessary to affect the many reported stem cell-based therapeutic outcomes in a variety of experimental models and clinical trials.

## 1. Introduction

Stem cells or progenitor cells are presently our best candidate therapeutic to treat intractable degenerative or genetic diseases through their capacity to engraft, differentiate, and generate new healthy cells to replace injured or diseased cells. This is best evidenced by the clinical success of hematopoietic stem cells as used in bone marrow transplantation to re-populate the recipient’s hematopoietic compartment with donor cells and treat a myriad of diseases such as cancer and genetic blood diseases such as thalassemia. Hence, the discovery of pluripotent stem cell (PSC) and non-hematopoietic tissue stem cells such as mesenchymal stem cell (MSC), neural stem cell (NSC), endothelial progenitor cell (EPC), or cardiac progenitor cells (CPC) has generated much optimism that non-hematopoietic diseases could also be similarly treated by replacing diseased cells with newly generated cells from pluripotent or tissue stem cells. Preliminary animal studies have demonstrated that many pluripotent or tissue stem cells have the potential to reduce the severity of many intractable degenerative or genetic non-hematopoietic diseases [1,2,3,4,5]. In fact, many of these stem or progenitor cells are being tested in clinical trials to treat many different diseases such as acute myocardial infarction (AMI), liver damage, ischemic kidney failure or stroke, amyotrophic lateral sclerosis (ALS), spinal cord injury, graft-*versus*-host disease (GVHD), *etc*. (Available online: http://www.clinicaltrials.gov).

### 1.1. Stem Cells

Stem cells (SCs) are generally defined as undifferentiated renewable cells that can differentiate into tissue-specific cell types with specialized functions. Currently, stem cells are divided into embryonic or “pluripotent” stem cells, and non-embryonic “somatic”, “adult” or “tissue” stem/progenitor cells. Embryonic stem cells (ESCs) are pluripotent stem cells derived from the inner cell mass of a blastocyst, an early-stage preimplantation embryo [6], and they are distinguished by two distinctive properties: pluripotency and the ability to replicate indefinitely. They are able to differentiate into the more than 200 cell-type derivatives of the three primary germ layers: ectoderm, endoderm, and mesoderm. Adult stem/progenitor cells, also known as somatic stem cells, are undifferentiated cells found throughout the body in juveniles as well as adults. These cells have large proliferative capacity and lineage-restricted differentiation potential, and could regenerate and contribute to physiological cell turnover [7,8,9,10]. To date, a large number of adult stem cells have been identified and isolated, and many have been reported to elicit therapeutic efficacy in animal studies and clinical trials. Some of these adult stem cells are MSCs, EPCs, CPCs, and NSCs. MSCs are multipotent stromal cells that can differentiate into a variety of mesodermal cell types [11] such as osteoblasts, chondrocytes, and adipocytes. They are also the most used stem cell type in clinical trials, primarily because they are multipotent, can be easily isolated from adult tissues, and have a large *ex vivo* expansion capacity [12]. EPCs, a subset of bone marrow-derived cells, are generally defined as circulating cells that express cell surface markers similar to those expressed by vascular endothelial cells, adhere to endothelium at sites of hypoxia/ischemia, and participate in new vessel formation [13,14]. CPCs are resident cardiac progenitor cells that are postulated to be derived from bone marrow or the embryonic cell population. They are thought to contribute to the physiological turnover of cardiac myocytes and vascular endothelial cells [15,16]. NSCs are self-renewing, multipotent cells that could be isolated from the fetal and adult brain, and have the potential to differentiate into neurons, astrocytes, and oligodendrocytes [17].

The use of embryonic *versus* adult stem cells for cell-based regenerative therapies has its own unique advantages and disadvantages [18]. Unlike adult stem cells whose *ex vivo* expansion capacity and differentiation potential are limited, embryonic stem cells have unlimited *ex vivo* expansion capacity and the potential to differentiate and replace almost every cell type in the adult body. However, adult stem cells are technically more amenable to our present regulatory framework and are ethically less controversial. In addition, the risk of immune rejection could be greatly reduced as adult stem cells could be harvested from the patient’s own body for *ex vivo* expansion and transplantation [19,20]. Their limited differentiation potential also mitigates the risk of forming aberrant or inappropriate tissues that could be deleterious, e.g., the formation of hard bone tissue in soft tissues like the brain. As such, the use of adult stem cells as therapeutic agents far exceeds that of ESCs and is currently being tested in the clinic against a large variety of disease indications.

### 1.2. Therapeutic Stem Cell Extracellular Vesicles (EVs)

Of the stem cells that are currently in clinical trials, the most widely used cell type is MSC and the other cell types are EPC, NSC and CPC (Available online: http://www.clinicaltrials.gov). The use of stem cells as therapeutics is often rationalized on their differentiation potential to generate replacement cell types. However this differentiation rationale was found to be increasingly inadequate, particularly for MSC which, being the widely used cell type, is also the best scrutinized. There are presently sufficient MSC studies to support an alternative proposal that MSC exerts its therapeutic effects through a secretion, and not a differentiation mechanism [12,21]. In many studies where functional improvement was reported after MSC transplantation, it was observed that migration, engraftment, and differentiation of MSCs at the sites of injury were rare [22,23,24], and involved <1% of transplanted cells [12]. It was also observed that migration of transplanted MSCs to the injured tissue is not necessary for efficacy [25,26,27,28].

The hypothesis that stem cells could exert therapeutic activity through their secretions is highly plausible as stem cell secretions are known to include many biologically potent molecules such as growth factors, cytokines, chemokines, and bioactive lipids that could elicit wide-ranging physiological effects [29]. This hypothesis was first validated for MSCs simply because they are the most studied stem cell type in therapeutic applications [21]. MSC-conditioned culture medium alone has been reported to recapitulate the efficacy of MSCs in cardioprotection [30,31,32], renal tubular cell survival [33], protection against fulminant hepatic failure [34,35], and immunomodulatory activity to alleviate immune disease [36]. However, it is unlikely that the capacity of MSCs in ameliorating complex and diverse tissue injuries such as myocardial ischemia/reperfusion injury or graft*-versus*-host disease could be attributed to a single molecular factor (reviewed [37,38]). Hence, EVs with their large and complex cargo of lipids, proteins, and RNAs are more likely candidates (reviewed [37,38]), and many stem cells are known to secrete EVs. Beside MSCs [39], ESCs [40], EPCs [41], NPCs [42], and CPCs [43] also secrete EVs. Some of the characteristics of EVs are comprehensively discussed in this focus edition by Kalra *et al.* [44].

With increasing evidence that EVs are major mediators of intercellular communication in many cell types [45,46], it is likely that EVs also perform similar functions for stem cells. As such, they would be expected to be significant in the hypothesis that stem cells exert therapeutic activity through their secretions by communicating therapeutic signals from stem cells to recipient cells to initiate repair and regeneration. The first therapeutically efficacious EVs secreted by stem cells were reported in 2009 when Bruno *et al.* reported that 180 nm MSC-derived microvesicles protect against acute tubular injury [39]. We subsequently reported that the smaller MSC-derived exosomes with a hydrodynamic radius of 55–65 nm also protect against acute myocardial ischemia/reperfusion injury [38,47], enhance wound healing [48], alleviate GVHD [49], reduce renal injury [50], and promote damaged hepatic regeneration [51]. Other groups have also reported the potential efficacy of MSC exosomes or EVs in treating other disease indications. For example, Xin *et al.* reported that MSC-derived exosomes promote neural plasticity and functional recovery in stroke via the transfer of miR-133b [52,53]. Katsuda *et al.* reported that human adipose MSC-derived exosomes contain functional neprilysin, a major β-amyloid peptide-degrading enzyme and, thus, have the potential to reduce the pathological accumulation of β-amyloid peptide in Alzheimer’s disease [54]. MSC-derived EVs were also found to protect against hypoxia- and endotoxin-induced lung injury [55,56]. More recently, EVs from CPCs have also been reported to be efficacious in cardiovascular disease [43,57,58,59]. EPC was reported to secrete EVs as early as 2007 [41], and these EVs enhance vascularization of xenotransplanted human islets in mice, alleviate renal ischemia/reperfusion injury in rats, and induce neovascularization in a murine model of hindlimb ischemia [60,61,62]. NSCs were first reported to secrete EVs in 2005 [42] and were shown in 2013 to promote neural plasticity and functional recovery after treatment of stroke. Together, these studies demonstrated that eutherapeutic outcomes of stem cell-treated tissue injury could be mediated by EVs [43,63,64,65] (as summarized in Table 1). Camussi *et al.* proposed that these EVs could potentially be home to target cells through receptors present on their surface such that, upon internalization, the cargo of EVs is loaded into diseased or injured target cells to initiate tissue repair and regeneration [66].

## 2. Mechanisms Underlying the Therapeutic Potential of Stem Cell EVs

**Figure 1:**
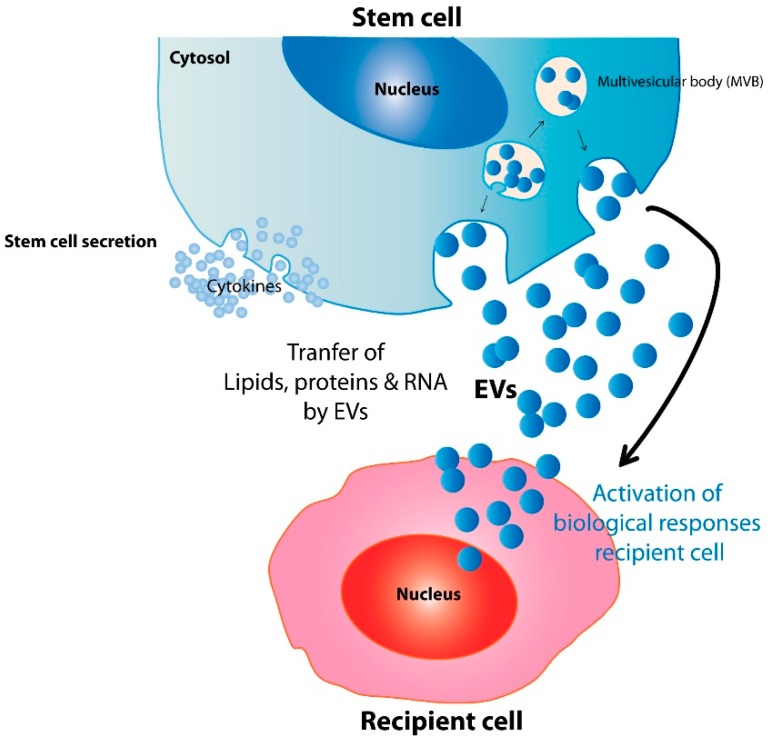
A proposed model for mechanisms underlying stem cell EV therapeutic potential. By delivery of lipids, proteins, and genetic information, stem cell-derived excellular vesicles (EVs) may biologically activate recipient cells to elicit relevant therapeutic effects.

In general, the functions of stem cell-derived EVs do not differ much from those found in EVs derived from non-stem cells. Essentially, stem cell EVs, like other EVs, function to transfer lipids, nucleic acids, and proteins from one cell to another to elicit biological responses, and it is well documented that EVs from stem cells could indeed elicit biological responses from recipient cells that are consistent with the contents of the EVs. For example, Ratajczak *et al.* [67] demonstrated that EVs from mouse ESCs enhanced survival and expansion of hematopoietic progenitor cells, and upregulated early pluripotent (Oct-4, Nanog, and Rex-1) and early hematopoietic stem cell (Scl, HoxB4, and GATA 2) markers. They attributed these effects to the presence of Wnt-3 protein and mRNA for pluripotent transcription factors in the mESC-derived EVs [67] (as illustrated in Figure 1 and Table 2).

Like ESC-derived EVs, EPC-derived EVs were also biologically active and their RNA cargo were implicated in EV-elicited biological or therapeutic responses such as angiogenesis [41] or protection against angiotensin II (Ang II)-induced cardiac hypertrophy and apoptosis [68]. Subsequent studies specifically implicated miR-126 and miR-296 [60,61] as the RNAs responsible for the angiogenic effect of EPC-derived EVs. CPCs are also known to exert therapeutic effects through RNAs delivered by their EVs. CPC-derived EVs protect against ischemia-reperfusion injury by delivering miR-451 to injured cardiomyocytes [57] or miR-146a to recapitulate the regenerative and functional effects of CPC transplantation [58]. Recently, it was suggested that grafted NSCs communicate with the host immune system by inducing interferon gamma signaling through EV-associated IFN-γ/Ifngr1 complexes [69] (as illustrated in Figure 1 and Table 2).

**Table 1:** Therapeutic role of stem cell EVs in various tissue injury models.

Author	Year	EV ^a^ Source	Disease or Assay Model	Therapeutic Effects	Ref.
Bruno *et al.*	2009	BM-MSC ^b^	Glycerol-induced acute kidney injury (AKI) in SCID ^c^ mice	Protect against acute tubular injury	[39]
Lai *et al.*	2011	ESC-MSC ^d^	Myocardial ischemia/reperfusion injury	Protect against acute myocardial ischemia/reperfusion injury	[38]
	2010				[47]
Zhang *et al.*	2014	UC-MSC ^e^	Rat skin burn model	Accelerate skin damage repair	[48]
Kordelas *et al.*	2014	BM-MSC	A therapy-refractory GVHD ^f^ patient	Improved the clinical GVHD symptoms significantly	[49]
Bruno *et al.*	2012	BM-MSC	Lethal cisplatin-induced AKI in SCID mice	Exert a pro-survival effect	[50]
Tan *et al.*	2014	ESC-MSC	Carbon tetrachloride (CCl4)-induced liver injury mouse model	Elicit hepatoprotective effects against toxicants-induced injury	[51]
Xin *et al.*	2012	BM-MSC	Middle cerebral artery occlusion and reperfusion model	Promote neural plasticity and functional recovery	[52]
	2013				[53]
Katsuda *et al.*	2013	ADSC ^g^	Co-culture of N2a cells with ADSCs	Decrease β-amyloid peptide (Aβ) levels in the N2a cells	[54]
Lee *et al.*	2012	UC-MSC	Murine model of hypoxic pulmonary hypertension	Exert a lung protection and inhibit pulmonary hypertension	[55]
Zhu *et al.*	2014	BM-MSC	*E. coli* endotoxin-induced acute lung injury (ALI) in mice	Restore lung protein permeability and reduce inflammation	[56]
Barile *et al.*	2014	CPC ^h^	Rat acute myocardial infarction (AMI) model	Inhibit cardiomyocyte apoptosis and improve cardiac function	[43]
Chen *et al.*	2013	CPC	Acute mouse myocardial ischemia/reperfusion (MI/R) model	Protect myocardium from acute MI/R injury	[57]
Ibrahim *et al.*	2014	CPC	Acute and chronic myocardial infarction model in SCID mice	Enhance angiogenesis and promote cardiomyocyte survival	[58]
Vrijsen *et al.*	2010	CPC	The *in vitro* scratch wound assay	Enhance migration of endothelial cells	[59]
Ranghino *et al.*	2012	EPC ^i^	Murine model of hindlimb ischemia in SCID mice	Induce neoangiogenesis and favor recovery	[60]
Cantaluppi *et al.*	2012	EPC	Rat acute kidney ischemia-reperfusion injury model	Protect the kidney from ischemic acute injury	[61]
Cantaluppi *et al.*	2012	EPC	Human islet transplantation model in SCID mice	Enhance insulin secretion, survival, and revascularization	[62]

^a^ EV: extracellular vesicles; ^b^ BM-MSC: bone marrow-derived mesenchymal stem cell; ^c^ SCID: severe combined immunodeficiency; ^d^ ESC-MSC: embryonic stem cell-derived mesenchymal stem cell; ^e^ UC-MSC: umbilical cord-derived mesenchymal stem cell; ^f^ GVHD: graft-*versus*-host disease; ^g^ ADSC: adipose tissue-derived mesenchymal stem cell; ^h^ CPC: cardiac progenitor cells; ^i^ EPC: endothelial progenitor cells.

**Table 2:** Examples of mechanisms underlying stem cell EV therapeutic potential.

EV Source	EV-Associated Active Contents	Biological Activities	Ref.
ESC	Wnt-3 protein and mRNA	Enhance hematopoietic progenitor cell survival and upregulate Oct-4, Nanog, Rex-1, Scl, HoxB4, and GATA 2	[67]
EPC	miR-126, miR-296	Angiogenesis or protection against angiotensin II-induced cardiac hypertrophy and apoptosis	[41,60,61,68]
CPC	miR-451, miR-146a	Protect against cardiac ischemia-reperfusion injury and recapitulate the regenerative and functional effects	[57,58]
NSC	IFN-γ/Ifngr1 complexes	Induce interferon gamma signaling	[69]
MSC	RNA/protein cargo	Protect against acute tubular injury and myocardial ischemia-reperfusion injury	[37,39,47,70,71,72,73]

The earliest stem cell reported to exert therapeutic effects through EVs is MSC, where MSC-derived EVs reportedly protect against acute tubular injury through their RNA cargo [39]. MSC-derived EVs also protect against myocardial ischemia-reperfusion injury [47]. Based on the cargo load of EVs [37,70,71], and the proteomic changes in the heart during myocardial ischemia-reperfusion injury [72], we hypothesize that MSC-derived EVs protect heart tissues against the injury by proteomic complementation to compensate for the proteomic alterations in myocardial ischemia-reperfusion injury and restore ATP production and induce survival signaling [73] (as illustrated in Figure 1 and Table 2).

We had also derived and characterized human fetal MSC and umbilical cord MSC as an alternative cell source for large-scale production of therapeutic cardioprotective EVs [74,75]. Consistent with the well-documented observation that the therapeutic efficacy of MSC is inversely correlated with the developmental stage of the donor, this correlation extended to the EVs. We observed that cord-derived MSC produced the least amount of therapeutic EVs, followed by fetal- and then ESC-derived MSC, suggesting that the inverse correlation between the therapeutic efficacy of MSC and the developmental stage of the donor is underpinned by the rate of EV production [75].

## 3. Conclusions

Stem cell EVs exert their therapeutic potential through the transfer of biologically active molecules in their vesicular cargo, which includes proteins, bioactive lipids, mRNA, and microRNA. The diversity of this EV cargo provides a rationale for the many reported stem cell-based therapeutic outcomes. There is now ample evidence of the effective recapitulation of the therapeutic efficacy of stem cells by their secreted EVs. This renders stem cell-derived extracellular vesicles a compelling alternative off-the-shelf, cell-free therapeutic modality that could be effective, safer, and cheaper. However, realizing this promising therapeutic modality of stem cell EVs would require extensive testing to validate their safety and efficacy.

For further information on the basic properties of EVs, their involvement in neurodegenerative and malignant diseases, their role in cell-cell communication, their potential as drug delivery vehicles, *etc.*, the reader is referred to the various reviews in this focus edition [44,76,77,78,79].
